# Giant hydatid cyst of the brain: Intact cyst removal in 8-year-old child

**DOI:** 10.1016/j.ijscr.2023.108172

**Published:** 2023-04-11

**Authors:** Amir Abbas Ghasemi, Haadi Mohammadzade, Roozbeh Mohammadi

**Affiliations:** a Urmia University of Medical Sciences, Ershad BLVD, Imam Khomeini Hospital, Urmia, Iran; b Urmia University of Medical Sciences, Iran

**Keywords:** Hydatid cyst, Brain, *Echinococcosis*, Surgery

## Abstract

**Introduction and importance:**

*Cystic echinococcosis* (*hydatidosis*) in humans is an infectious disease caused by tapeworms of *Echinococcus* genus. Brain involvement is rare. The best treatment is surgery and intact cyst removal is mandatory to prevent recurrence and possible anaphylactic reaction.

**Case presentation:**

An 8-year-old girl presented with a 1-month history of malaise, headache and vomiting. On Physical examinations, she was disoriented with bilateral papilledema. Brain CT scan and MRI revealed a well-defined cystic mass in left temporo-parieto-occipital region with considerable midline shift without perilesional edema. The patient was diagnosed with brain hydatid cyst and underwent surgical excision of the cyst without rupture.

**Clinical discussion:**

Surgery is the most important part of intracranial hydatid cyst treatment, and surgeons should make every effort to remove them in toto without spillage. We hence decided to perform surgery and necessary precautions to prevent rupture and dissemination of hydatid were taken during the surgery.

**Conclusion:**

A neurosurgeon has to bear in mind brain hydatid cyst in the differential diagnosis of cystic cerebral lesions especially in children from rural areas. The hydrodissection technique is the gold standard for the surgical treatment of cerebral hydatid cyst disease. It can also be effectively applied to the treatment of giant cerebral hydatid cyst disease without rupturing the cyst.

## Highlights

• Hydatidosis in humans is an infectious disease caused by tapeworms of *Echinococcus* genus.
• Brain involvement is rare.
• The best treatment is surgery.
• Intact cyst removal is mandatory to prevent recurrence and possible anaphylactic reaction.

## 1 Introduction

*Cystic echinococcosis* (*CE*) also referred to as *hydatid cyst* is an infection caused by larval stage of the dog tapeworms *Echinococcus granulosus* and *Echinococcus multilocularis* [1]. Human acquire the infection by accidental ingestion of Taeniid eggs. Humans as intermediate hosts may have different organ involvement such as liver (the most common), lung, muscles, heart, spine and brain [2]. The most common parasitic infection of the brain is *hydatid cyst*, but primary cerebral *hydatid cyst* is rare (1–2 % of all cases) [3]. Cerebral hydatid cyst is usually diagnosed during childhood and is often solitary. Multiple cerebral *hydatid cysts* usually result from dissemination secondary to spontaneous, traumatic or surgical rupture of a primary cyst. Although CE may be located anywhere in the brain, it is most frequently found in both hemispheres, particularly in the middle cerebral artery territory with the parietal lobe being the most common site. Very rarely the cysts are located in the posterior cranial fossa or ventricles. In patients with cerebral hydatid disease, concurrent hepatic, pulmonary and other organ involvement has been reported. Brain involvement often presents as a well-defined isolated cystic lesion in the territory of the middle cerebral artery [4]. The best treatment modality is surgery and the aim of the surgery is total extirpation of the cyst without rupturing the cyst wall [5]. In this report, we illustrate the case of an 8-year old girl with brain *hydatid cyst* in left parieto-occipital region who was treated in our hospital via left parieto-occipital craniotomy. The cyst was removed totally intact using Dowling's technique of hydrodissection. This case report has been reported in line with the SCARE Criteria [6].

## 2 Presentation of case

**Fig. 1:**
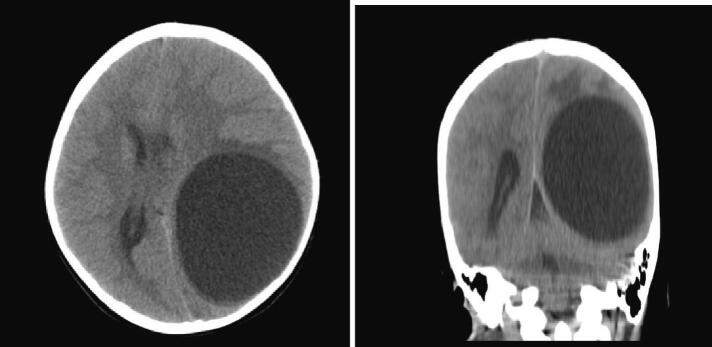
Axial and coronal brain CT Scan showing cystic lesion in left temporo-parieto-occipital region.

**Fig. 2:**
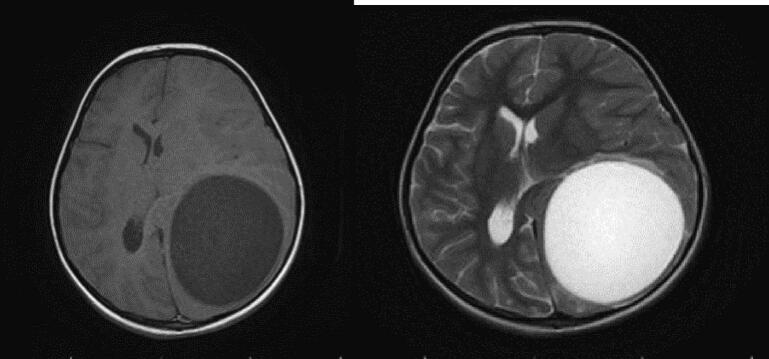
Axial T1W and T2W MRI showing a spherical and well-defined, smooth, thin walled, homogeneous cystic lesion with signal similar to the cerebrospinal fluid without septations.

**Fig. 3:**
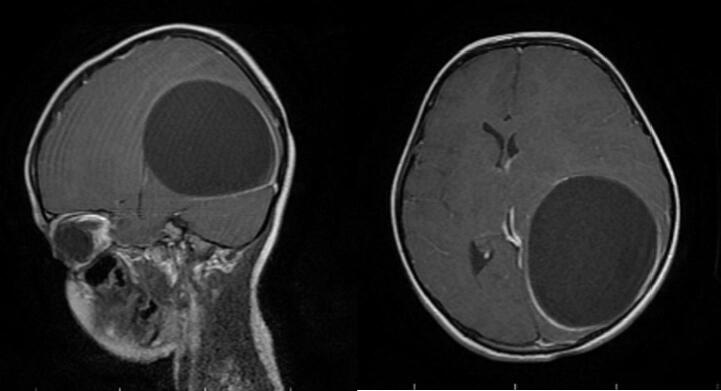
Sagittal and Axial T1W MRI with contrast showing posterior and lateral walls enhancement.

**Fig. 4:**
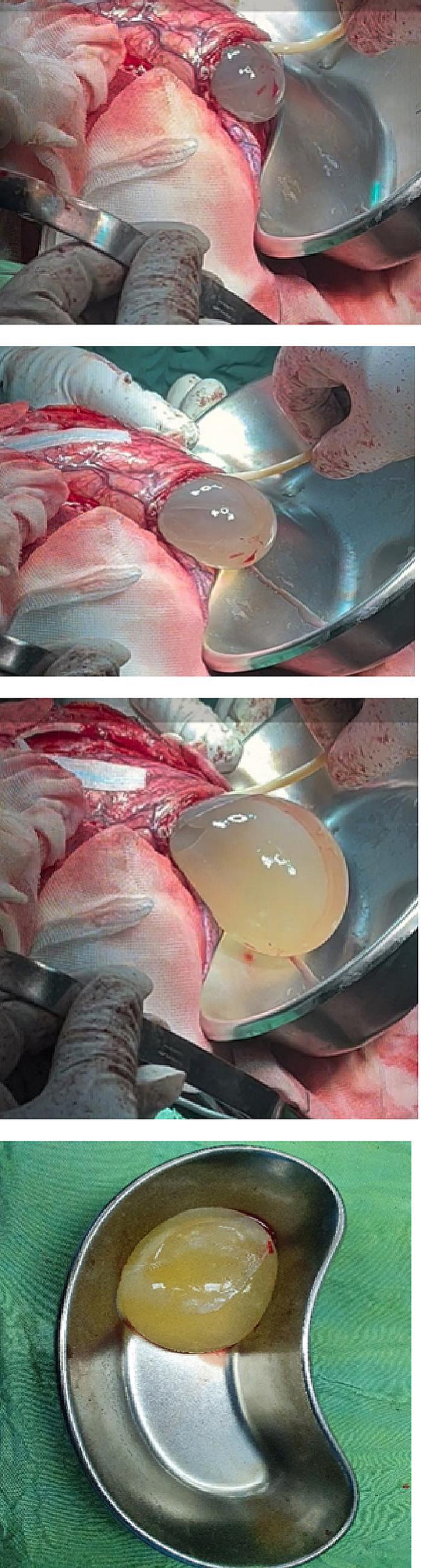
Gradual separation and delivery of the brain hydatid cyst with hydrodissection.

An 8-year-old girl presented with a 1-month history of malaise, headache and vomiting prior to admission. She lived in rural areas of west Azerbayjan province of Iran. On Physical examinations, she was disoriented with bilateral papilledema. The results of routine laboratory blood tests were nonspecific. Serologic test results for CE were negative. CT Scans of the Chest and Abdomen failed to reveal any associated *hydatid cyst* in the lungs and abdomen. Brain CT scan revealed a large, well-defined cystic lesion in the left temporo-parieto-occipital region with considerable mass effect (Fig. 1). Brain MRI demonstrated a spherical and well-defined, smooth, thin walled, homogeneous cystic lesion with signal similar to the cerebrospinal fluid without septations (Fig. 2). There was no perilesional edema. The cyst showed posterior and lateral walls enhancement after gadolinium injection (Fig. 3). Based on imaging findings the diagnosis of brain hydatid cyst was given. The patient was brought to the operating room and after full preparation, the patient underwent left temporoparieto-occipital craniotomy. Dura was opened circumferentially away from the cyst, and the cyst was seen after a small cerebrotomy. The plane between cortex and cyst wall was delineated. Catheter tip introduced in this plane irrigation with saline was started. To facilitate removal, the cyst was made gravity dependent by tilting head end of the table down by 10°–20°. Gradually, the cyst started separating from the parenchyma and was eventually separated from the cortex as a whole without rupture and delivered in a bowl (Fig. 4). Pathological examination of the surgical specimen was consistent with hydatid cyst. Postoperative recovery was uneventful, and the patient was discharged on albendazole (dose: 15 mg/kg/d in two divided doses) for 3 months. The 6 months follow-up showed no recurrence of cyst.

## 3 Discussion

Hydatid disease is an ancient disease and even was known to Hippocrates [7]. This parasitosis is caused by the larva *of Echinococcus granulosus*. The *adult Echinococcus granulosus* lives in the small bowel of the primary hosts (carnivorous animals) and produces Taeniid eggs that are released from the intestinal tract into the environment [8], [9]. After oral uptake of eggs by a secondary host, a larval stage, *the metacestode*, develops in internal organs, especially the liver and lungs [10]. In these organs, the oncosphere develops into a cyst that enlarges gradually, producing protoscolices and daughter cysts that fill the cyst interior. Humans are infected incidentally and usually in childhood by ingestion of the ova. Brain hydatids account for 2 % of all hydatid lesions that are treated. The rate of growth of the cyst varies in different structures—faster in soft tissues such as brain and liver and slower in hard tissues, notably in the bones [11].

*Hydatidosis* has a worldwide distribution with annual incidence rate of 1–200 per 100,000. It is endemic in many sheep and cattle-raising geographic areas including Mediterranean countries, the Middle East, Eastern Europe and South America. In Iran, the *hydatidosis* is being actively transmitted and its annual incidence rate is estimated 0.61 per 100,000 [12].

Cerebral involvement is extremely rare, being seen in 2-3% of systemic disease and forming 2% of all space occupying lesions of the brain. This rarity, coupled with nonspecific symptoms, necessitates a high index of clinical suspicion and thus presents a diagnostic challenge [14].

It is observed more often in children and young adults and several case series reported a slight male prevalence [15]. They can be seen in any part of the brain but are usually supratentorial and located in the middle cerebral artery territory, most commonly the parietal lobe [16]. Sometimes large, single cysts can be observed in the frontoparietotemporal region. The less common sites of involvement are cerebellum, pons, ventricles, cavernous sinus [17].

Brain hydatid cysts are usually single and occurrence of multiple cysts is very rare [18].

The patients are usually asymptomatic or their symptoms are nonspecific since growth of the cysts is generally slow and therefore clinical manifestations tend to be nonspecific due to compression of involved tissues. The diagnosis depends on clinical suspicion, typically based on a history of living in, or having travelled to, an endemic area and is evaluated with imaging [13].

The signs and symptoms are nonspecific and the most common ones are reported to be headache, papilledema, and vomiting; however, any symptom due to increased intracranial pressure can be seen. Focal symptoms like hemiparesis, seizures, gait, or Vision Problems can be observed depending on the size and location of the cyst [19].

In *cerebral hydatid cyst* both computerized tomography (CT) and MRI reveal a spherical cystic lesion with well-defined borders, a smooth thin wall with or without septation or calcification. The cyst wall is iso- or hyperdense with respect to the cerebral parenchyma on unenhanced CT and usually shows a rim of low intensity in both T1W and T2W images. Daughter cysts, if present, are considered pathognomonic but are rarely seen. Wall calcification is seen in less than 1 % of cases. Mass effect, with compression of midline structures and the ventricles, is a common finding, but surrounding edema and rim enhancement are generally not seen in uncomplicated cases. Thin rim of peripheral enhancement can be visualized due to fibrous capsule or secondary to superadded infection [20].

The differential diagnosis of an intracerebral *hydatid cyst* with mainly typical characteristics includes supratentorial cystic lesions like arachnoid cysts, cystic tumors, abscess, and porencephalic cysts. Arachnoid cysts are not spherical, porencephalic cysts are usually connected to the ventricular system and neither are entirely surrounded by brain tissue, cystic tumors usually have solid components that are enhanced after contrast injection, and abscesses typically demonstrate rim enhancement and oedema of surrounding white matter.

As a conclusion, imaging is useful, but not always conclusive, in diagnosing brain hydatid cyst. CT detects calcification in the lesion better than MRI, whereas MRI is better for assessment the exact location and anatomic relationships of the lesion. Nevertheless, in some cases, despite use of advanced imaging techniques, the diagnosis is still problematic. *Hydatid cyst* should be considered especially with supratentorial cystic lesions in young male patients with a history of living in an endemic area or having contact with farm dogs and cattle.

The therapeutic options that can be used in cases of hydatid cysts include: surgical excision, PAIR (puncture, aspiration, injection of protoscolicidal agent and reaspiration), chemotherapy with anthelmintic agent (Albendazole), and conservative treatments, i.e., “watch and see”, which are useful in cases of inactive cysts. Small, heavily calcified cysts can be considered inactive or dead and can only be monitored periodically.

Surgery is the mainstay of intracranial *hydatid cyst* treatment, and surgeons should do their best to remove them in toto without spillage. The most common complication is a rupture of the cyst into the subarachnoid space which leads to widespread dissemination followed by severe inflammatory or anaphylactic response. Dowling's technique is the most accepted method nowadays. It requires a great osteoplastic flap, cautious handing during all the surgical steps, wide incision over the cyst, sloping the head of the table downward, and injecting saline between the cyst and the brain. In our case, we opted for surgical treatment, removed the cyst in toto and obtained a good outcome.

## 4 Conclusion

A neurosurgeon has to bear in mind brain hydatid cyst in the differential diagnosis of cystic cerebral lesions especially in children from rural areas. The hydrodissection technique is the gold standard for the surgical treatment of cerebral hydatid cyst disease. It can also be effectively applied to the treatment of giant cerebral hydatid cyst without rupturing the cyst.

## Consent

Written informed consent was obtained from the patient's parents/legal guardian for publication of this case report and accompanying images. A copy of the written consent is available for review by the Editor-in-Chief of this journal on request.

## Ethical approval

We obtained approval from the ethics committee of Urmia University of Medical Sciences, West Azerbaijan, Iran (Code: IR.UMSU.REC.1401.308).

## Funding

The authors have not received any funding for their paper.

## Author contribution

Design of the article the acquisition, analysis, or interpretation of data for the article (Amir Abbas Ghasemi, Haadi Mohammadzade).

Data collection (Haadi Mohammadzade, Roozbeh Mohammadi).

Writing the paper (Amir Abbas Ghasemi).

## Guarantor

Amir Abbas Ghasemi.

## Research registration number

Not applicable.

## Declaration of competing interest

The authors had no conflicts of interest.
